# Supplementary material for: Novel Biomarkers for Early Detection of Acute Kidney Injury and Prediction of Long-Term Kidney Function Decline after Partial Nephrectomy
Source: Biomedicines. 2023 Mar 28;11(4):1046. doi: 10.3390/biomedicines11041046 (PMC10135876; doi:10.3390/biomedicines11041046)
Supplement: Supplementary file 1 [file biomedicines-11-01046-s001.zip › Supplementary methods.pdf]

Supplementary methods

1.1. Definitions of AKI and CKD

Current guidelines from KDIGO recommend using estimated GFR from creatinine (eGFR) and not simple sCr values, because it allows a more accurate estimation of kidney function. The eGFR was calculated using Chronic Kidney Disease Epidemiology Collaboration equations (CKD-EPI creatinine formula)[1] which more appropriately categorized individuals with respect to long-term clinical risk.[2]

The baseline preoperative eGFR has been calculated as the average of two different sCr values detected in between the two months before surgery to minimize the effects of great variability of sCr values. In fact, inaccurate determination of baseline kidney function can misclassify AKI, and use of the admission sCr value as baseline significantly underestimate the AKI incidence[3] and a developing consensus favours the use of an average sCr 7–365 days up to admission.[4,5]

According to the Kidney Disease Improving Global Outcomes (KDIGO) guidelines,[6] patients were classified as CKD if they had preoperative GFR less than 60 mL/min/1.73m^2 and/or persistent proteinuria.

According to the KDIGO recent consensus definition of AKI accepted for clinical practice,[7] AKI was defined as a serum creatinine increase ≥ 0.3 mg/dL within 48 hours, or a serum creatinine increase of 1.5 times the baseline value known to have occurred within the prior 7 days and/or with subsequent documented normalization. All types of AKI were included in the study, regardless of the underlying etiology.

1.2. Biomarkers assay and sample collection

In our study, the sCr, hourly urinary output and 8 different candidates were measured as AKI biomarkers. Molecules with a rapid response to injury were selected to better identify early AKI onset. Furthermore, with the ultimate aim of investigating the underlying etiopathogenetic

mechanisms of AKI, we selected a panel of AKI biomarkers representative of the different segments of the nephron (which are differently affected in relation to the nature of the damage) (**Figure 2**). Consequently, we selected for this study the most promising biomarkers in the NSS setting, as identified in a recent review,[8] also including two novel promising biomarkers never tested in NSS patients (NephroCheck® test and kineticGFR) and two biomarkers of pure glomerular damage (albuminuria and acanthocytes in urinary sediment), in order to understand the most damaged renal compartment in renal surgery.

The creatinine assay in serum and urine was carried out through an enzymatic method. Proteinuria was measured through a red color Pirogallolo methodology, using the instrumentation of clinical chemistry Vista Siemens, while albuminuria was measured through a colorimetric method with Beckman Coulter instrumentation. The assay of these analytes was performed after the serum and urine sample was centrifuged at 3500 rpm for 10 minutes. The evaluation of acanthocytes, in the urinary sediment, was carried out under phase contrast microscope after slide preparation after centrifugation of the urine sample at 2000 rpm for 10 minutes. All measurements were made in our institution's biochemistry department.

Serum and urinary NGAL were collected in EDTA anticoagulated tubes and were immediately analyzed using the NGAL Test™ (BioPorto Diagnostics, Gentofte, Denmark), which has been previously validated and compared to different commercially available NGAL assays.[9]

Urine tissue inhibitor of metalloproteinase 2 (TIMP-2) and insulin-like growth factor binding protein 7 (IGFBP7) are considered two new promising biomarkers. TIMP-2 seems to be a biomarker dedicated to distal tubular cells, whereas insulin-like growth factor-binding protein 7 (IGFBP7) secretion has been found in proximal tubule cells (**Figure 2**). The product of urinary [TIMP-2]*[IGFBP7] appeared in several studies more sensitive than sCr to diagnose and describe AKI severity,[10] but their role in patients undergoing NSS is unknown. Fresh urine samples taken during the last hour of collection were placed in dry tubes and were immediately analyzed for [TIMP-2]*[IGFBP7] using the NephroCheck® Test point-of-care fluorescence immunoassay (Astute Medical, San Diego, CA, California). The ASTUTE140 Meter automatically multiplied the

concentrations of the two biomarkers together and divided this product by 1,000 to report a single numerical test result with units of (ng/ml)2 /1,000. Based on the Sapphire and Opal studies,[11,12] the manufacturer suggests using >0.3 as moderate risk and >2.0 as high risk of severe AKI. The test cutoff is a score based upon the product of the concentration in the urine of these two proteins divided by 1000.

Serum cystatin C was measured by an immunoassay in nephelometry. A relative increase ≥ 10% from baseline of serum cystatin C concentration, within 24 hours after renal damage, was previously described as the best cystatin C cutoff for the early identification of AKI in several studies.[13,14] However, the overall accuracy of serum cystatin C for the identification of AKI was demonstrated to be high, as showed by a meta-analysis of 30 prospective cohort studies. [10]

In addition, kinetic eGFRs (kineticGFR) were calculated as estimates of the creatinine clearance within a discrete time frame calculated from two sCr measurements at different time points after PN ($kGFR_{preop-4h}$: $kGFR_{preop-12h}$; $kGFR_{preop-24h}$; $kGFR_{4h-12h}$; $kGFR_{4h-24h}$; $kGFR_{12h-24h}$).[15] kGFR allow estimation of rapidly changing GFR, reflecting dynamic changes in renal function between preoperative and postoperative. Even when compared with different biomarkers, kGFR appeared as an early and specific marker of AKI[16] but its role in patients undergoing NSS is unknown.

1.3. Anaesthesiology protocol

For each patient, general anaesthesia was induced with Midazolam 0,03 mg/kg, Fentanyl 2-3 mcg/kg and Propofol 1,5 - 2,5 mg/kg, according with established Guidelines.

A total intravenous anaesthesia was maintained through Propofol and Remifentanil in target-controlled infusion. Processed elecroencephalographic (pEEG) monitoring was employed for each patient. Cisatriacurium 0,15 mg/kg or Rocuronium 0,6 - 1 mg/kg were used as muscle relaxant during Train of four (TOF) monitoring.

Standard monitoring was achieved during anaesthesia, according to National Society of Anaesthesiologists (SIAARTI) Guidelines. In all cases, if no drug allergy was reported preoperatively, ceftriaxone 2g was administrated intravenously as perioperative antibiotic prophylaxis.

Ventilation was performed following successful endotracheal intubation to maintain EtCO2 concentration between 35 and 45 mmHg.

Fluid maintenance was obtained with crystalloid ranging from 3 to 6 ml/kg/h (according with dynamic parameters of fluid responsiveness); no plasma expander and mannitol were administrated. Body temperature was maintained intraoperatively (35.5-36.5°C) through active external rewarming.

Post-operative analgesia was obtained intravenously as follow: morphine 0,1 mg/Kg after tumor resection during the renal reconstructive phase (approximately 45 minutes before the end of surgery) and paracetamol 1 g 30 minutes before the end of surgery; then, Paracetamol 1 g intravenous twice a day and morphine in Patient Controlled Analgesia (PCA, bolus of 1 mg, 20 minutes lock-out) was used for the first 24 hours after surgery.

References

1. Levey AS, Stevens LA, Schmid CH, et al (2009) A new equation to estimate glomerular filtration rate. Ann Intern Med 150(9):604-12.
2. Matsushita K, Mahmoodi BK, Woodward M, et al (2012) Comparison of risk prediction using the CKD-EPI equation and the MDRD study equation for estimated glomerular filtration rate. JAMA 307:1941–1951.
3. Siew ED, Matheny ME, Ikizler TA, et al (2010) Commonly used surrogates for baseline renal function affect the classification and prognosis of acute kidney injury. Kidney Int 77(6):536-42.
4. Siew ED and Matheny ME (2015) Choice of reference serum creatinine in defining acute kidney injury. Nephron 131(2):107-12.
5. Arnold J, Sims D, Gill P, et al (2019) Acute kidney injury calculated using admission serum creatinine underestimates 30-day and 1-year mortality after acute stroke. Clin Kidney J 13(1):46-54.
6. KDIGO AKI Work Group (2012) KDIGO clinical practice guideline for acute kidney injury.

Kidney Int Suppl 2: 1-138.

7. Bellomo R, Ronco C, Kellum JA, et al (2004) Acute renal failure - definition, outcome measures, animal models, fluid therapy and information technology needs: the Second International Consensus Conference of the Acute Dialysis Quality Initiative (ADQI) Group. In: Critical care (London, England).

8. Antonelli A, Allinovi M, Cocci A, et al (2020) The Predictive Role of Biomarkers for the Detection of Acute Kidney Injury After Partial or Radical Nephrectomy: A Systematic Review of the Literature. Eur Urol Focus 6(2):344-353.

9. Lippi G, Aloe R, Storelli A, et al (2011) Evaluation of NGAL Test™, a fully-automated neutrophil gelatinase-associated lipocalin (NGAL) immunoassay on Beckman Coulter AU 5822. Clin Chem Lab Med 50(9):1581-4.

10. Liu Y, Chen KH, Chen SQ, et al (2017) Predictive value of post-procedural early (within 24 h) increase in cystatin C for contrast-induced acute kidney injury and mortality following coronary angiography or intervention. Oncotarget 8(65):109762-109771.

11. Kashani K, Al-Khafaji A, Ardiles T, et al (2013) Discovery and validation of cell cycle arrest biomarkers in human acute kidney injury. Crit Care 17(1):R25.

12. Zhang D, Yuan Y, Guo L, et al (2019) Comparison of urinary TIMP-2 and IGFBP7 cut-offs to predict acute kidney injury in critically ill patients. Medicine (Baltimore) 98:e16232.

13. Briguori C, Visconti G, Rivera NV, et al (2010) Cystatin C and contrast-induced acute kidney injury. Circulation 121(19):2117-22.

14. Yin L, Li G, Liu T, et al (2013) Probucol for the prevention of cystatin C-based contrast-induced acute kidney injury following primary or urgent angioplasty: A randomized, controlled trial. Int J Cardiol 167(2):426-9.

15. Yang C, Zhou J, Lu ZX, et al (2019) Simultaneous treatment of ureteropelvic junction obstruction complicated by renal calculi with robotic laparoscopic surgery and flexible cystoscope. World J Urol 37:2217–2223.

16. Dewitte A, Joannès-Boyau O, Sidobre C, et al (2015) Kinetic eGFR and novel AKI biomarkers to predict renal recovery. Clin J Am Soc Nephrol 10(11):1900-10.
