# Supplementary material for: Novel Biomarkers for Early Detection of Acute Kidney Injury and Prediction of Long-Term Kidney Function Decline after Partial Nephrectomy
Source: Biomedicines. 2023 Mar 28;11(4):1046. doi: 10.3390/biomedicines11041046 (PMC10135876; doi:10.3390/biomedicines11041046)
Supplement: Supplementary file 1 [file biomedicines-11-01046-s001.zip › Table S1.pdf]

Supplementary Table S1. Clinical and laboratory predictors of AKI at different postoperative time points, stratified by the occurrence of AKI

		tot	AKI	Non AKI	OR [CI95%]	p value	AUC [IC95%]
Serum creatinine	4 hr	1.03 (0.90; 1.26)	1.15 (0.98; 1.38)	0.92 (0.79; 1.08)	45.10 [1.6; 1269]	0.03	0.76 [0.60; 0.92]
	10 hr	1.04 (0.86; 1.41)	1.25 (1.04; 1.46)	0.90 (0.75; 1.21)	15.7 [1.36; 181.1]	0.03	0.75 [0.59; 0.92]
	24 hr	1.06 (0.88; 1.39)	1.40 (1.08; 1.50)	0.94 (0.84; 1.12)	61.7 [3.26;1168]	<0.01	0.83 [0.70; 0.97]
Cystatin C	4 hr	1.06 (0.84; 1.24)	1.10 (1.04; 1.30)	0.89 (0.79; 1.12)	19.52 [0.97;392.00]	0.05	0.73 [0.55; 0.90]
	10 hr	1.04 (0.86; 1.31)	1.19 (0.96; 1.39)	0.92 (0.84; 1.22)	8.07 [0.86; 75.87]	0.07	0.68 [0.50; 0.86]
	24 hr	0.99 (0.84; 1.23)	1.17 (0.98; 1.43)	0.92 (0.75; 1.10)	42.02 [1.77;998.89]	0.02	0.77 [0.61; 0.92]
Serum NGAL	4 hr	307.92 (242.48; 429.60)	372.71 (289.96; 463.99)	262.39 (204.12; 394.45)	1.00 [1.00; 1.01]	0.13	0.69 [0.51; 0.88]

	10 hr	304.74 (234.50; 545.14)	375.63 (300.04; 585.40)	268.28 (215.67; 351.65)	1.00 [1.00; 1.01]	0.07	0.75 [0.56; 0.93]
	24 hr	267.09 (192.98; 421.66)	473.92 (293.62; 525.65)	207.36 (184.88; 332.12)	1.01 [1.00; 1.02]	0.01	0.82 [0.64; 1.00]
Urinary NGAL	4 hr	0.0 (0.0; 93.6)	41.2 (0.0; 665.7)	0.0 (0.0; 25.8)	1.04 [0.99; 1.08]	0.09	0.71 [0.54; 0.87]
	10 hr	136.8 (0.0; 297.5)	177.5 (8.2; 1160.8)	74.3 (0.0; 214.0)	1.02 [1.00;1.03]	0.07	0.66 [0.49; 0.84]
	24 hr	244.9 (102.2; 480.5)	287.2 (108.6; 749.4)	234.3 (0.0; 462.7)	1.00 [0.99;1.01]	0.67	0.55 [0.35; 0.74]
NephroCheck® Test	4 hr	0.30 (0.08; 0.74)	0.64 (0.32; 1.07)	0.14 (0.04; 0.33)	3.50 [0.86;14.24]	0.08	0.78 [0.64; 0.93]
	10 hr	0.47 (0.20; 1.09)	0.63 (0.39; 1.36)	0.34 (0.11; 0.64)	1.50 [0.75; 3.00]	0.26	0.68 [0.51; 0.85]
	24 hr	0.30 (0.08; 0.88)	0.42 (0.16; 1.10)	0.21 (0.06; 0.84)	1.76 [0.46; 6.76]	0.41	0.59 [0.34; 0.84]
Proteinuria	4 hr	23.50 (14.00; 43.00)	24.50 (14.00; 66.00)	23.50 (15.00; 42.50)	1.00 [0.98; 1.02]	0.88	0.49 [0.28; 0.71]

	10 hr	23.00 (13.00; 49.00)	28.00 (14.50; 50.50)	22.00 (13.00; 38.00)	1.00 [0.98; 1.02]	0.76	0.53 [0.33; 0.73]
	24 hr	25.00 (14.00; 52.00)	31.50 (21.00; 48.00)	21.00 (14.00; 52.00)	1.00 [0.98; 1.02]	0.98	0.41 [0.21; 0.60]
Proteinuria/ Creatininuria	4 hr	0.51 (0.29; 0.76)	0.65 (0.32; 0.88)	0.42 (0.29; 0.64)	2.91 [0.28; 29.76]	0.37	0.59 [0.37; 0.80]
	10 hr	0.33 (0.23; 0.52)	0.32 (0.22; 0.49)	0.37 (0.28; 0.54)	1.59 [0.17; 15.30]	0.69	0.46 [0.27; 0.65]
	24 hr	0.39 (0.26; 0.57)	0.40 (0.21; 0.57)	0.36 (0.29; 0.52)	1.15 [0.12; 10.75]	0.90	0.46 [0.24; 0.67]
Albuminuria	4 hr	189.60 (70.00; 388.90)	352.30 (253.30; 602.90)	124.90 (62.60; 382.60)	1.00 [1.00; 1.01]	0.19	0.67 [0.44; 0.90]
	10 hr	150.45 (75.40; 256.40)	194.60 (80.00; 388.45)	132.30 (68.70; 222.70)	1.00 [1.00; 1.01]	0.23	0.57 [0.33; 0.80]
	24 hr	94.25 (66.20; 217.50)	84.60 (70.40; 281.20)	95.40 (66.20; 153.00)	1.00 [1.00; 1.01]	0.22	0.57 [0.35; 0.79]
Kinetic GFR	4 hr	52.9 (27.4;	22.6 (0.0;	66.6 (45.2;	0.96 [0.93;	0.004	0.82 [0.69; 0.96]

		70.7)	52.9)	77.2)	0.99]		
	10 hr	60.4 (38.8; 72.9)	37.2 (24.1; 61.1)	68.5 (56.7; 77.8)	0.94 [0.90; 0.98]	0.005	0.82 [0.68; 0.95]
