# Supplementary material for: Novel Biomarkers for Early Detection of Acute Kidney Injury and Prediction of Long-Term Kidney Function Decline after Partial Nephrectomy
Source: Biomedicines. 2023 Mar 28;11(4):1046. doi: 10.3390/biomedicines11041046 (PMC10135876; doi:10.3390/biomedicines11041046)
Supplement: Supplementary file 1 [file biomedicines-11-01046-s001.zip › Table S2.pdf]

**Table S2.** Role of biomarkers as predictors of eGFR decline at 24 months postoperatively

| | CC [IC95%] | p value |
|---|---|---|
| Serum creatinine 24h postoperatively | -0.25 (-0.53; 0.07) | 0.119 |
| Serum cystatin C 24h postoperatively | -0.22 (-0.51; 0.11) | 0.189 |
| Serum NGAL 10h postoperatively | -0.31 (-0.60; 0.05) | 0.082 |
| urinary NGAL 10h postoperatively | -0.41 (-0.65; -0.11) | 0.009 |
| urinary NephroCheck 4h postoperatively | -0.27 (-0.54; -0.05) | 0.096 |
| urinary NephroCheck 10h postoperatively | -0.47 (-0.69; -0.18) | 0.0023 |
| Proteinuria 10h postoperatively | -0.26 (-0.54; 0.07) | 0.11 |
| Albuminuria 24h postoperatively | -0.23 (-0.53; 0.11) | 0.178 |
| KineticGFR 4h postoperatively | 0.34 (0.02; 0.59) | 0.034 |
| KineticGFR 10h postoperatively | 0.29 (0.03; 0.56) | 0.07 |
